# Informing about the invisible: communicating en route air pollution and noise exposure to cyclists and pedestrians using focus groups

**DOI:** 10.1186/s12544-022-00571-0

**Published:** 2022-11-08

**Authors:** Heike Marquart

**Affiliations:** 1 Institute of Transport Research, German Aerospace Center (DLR) Berlin, Rudower Chaussee 7, 12489 Berlin, Germany; 2 Geography Department, Humboldt University Berlin, Unter den Linden 6, 10099 Berlin, Germany

**Keywords:** Air pollution, Noise pollution, Personal exposure, Feedback, Information, Environmental health literacy

## Abstract

Active mobility and public transport are considered beneficial for health and wellbeing and valuable for climate change mitigation. However, cyclists and pedestrians have high air pollution and noise exposure alongside traffic, which adversely impact health and wellbeing. The measured exposure can differ from the perceived exposure, hence, communicating en route exposure is crucial. Therefore, this study investigates how to communicate route-based exposure to cyclists and pedestrians and explores if exposure communication, e.g. via smartphones, is worthwhile for healthy and pleasant commute. It is investigated how exposure feedback influences the motivation to protect oneself and how exposure information should be designed. Three focus groups with 20 cyclists/pedestrians living in Berlin, Germany, were conducted. Based on Protection Motivation Theory and Environmental Health Literacy concept, (1) experiences and practices after recognizing exposure were discussed and (2) information needs and communication strategies were developed. The results reveal a feeling of helplessness regarding the ubiquity and uncertainty of pollution and a heightened threat appraisal. Anger, anxiety and rejection were stated. Making sense of pollution levels and protective alternatives were central. A healthy routing app, including also pleasant route factors, was desired. However, information provision was also denied. Participants argued the responsibility should not be left to the commuters and planning for exposed road users would be crucial. Information provision may not be worthwhile if planning authorities do not provide healthy alternatives. People-centered approaches for tackling air pollution and noise exposure en route should be investigated further.

## Introduction

Ambient air pollution and noise pollution are two of the leading environmental health risk factors in urban areas, also in Europe [1–3]. Air pollution can cause respiratory diseases or cardiovascular diseases, psychological distress and impact wellbeing [3–7]. Particulate matter, specifically PM2.5, is considered responsible for a high number of premature deaths every year in Europe [3] and is the fifth-ranking mortality risk factor globally [8]. Noise exposure can lead to annoyance, psychological stress and impacts physical health [2]. A major source of air pollution and noise pollution in cities is motorized road and rail traffic [9]. Traffic related air pollution and noise pollution is high in the urban core alongside road- and rail-traffic with high traffic volume and might be even reinforced by low air exchange [10, 11]. Cyclists and pedestrians, who move alongside road- and rail-traffic, are exposed to air pollution and noise pollution higher than when separated from motorized traffic [12]. Some studies estimate they inhale higher doses of air pollution than motorized transport commuters [13, 14]. Likewise, underground subway users are exposed to higher levels of particulate matter (PM2.5) than above ground [15]. The noise level that cyclists, pedestrians or public transport users are exposed to is frequently higher than of car users [10, 16].

Even though exposure and health impacts are evident, recent studies show that cyclists and pedestrians do not perceive their exposure as it is measured by sensors [17–19] and do not see them as an impediment to walk or cycle [20]. For example, a study in Leipzig, Germany, showed that over 80% of the surveyed cyclists underestimated their exposure to particulate matter and noise pollution [19]. When people are exposed to noise over a longer period of time they adapt to it and the annoyance decreases [21]. A recent review shows that in most studies perceived and measured pollution (not only in traffic) match, however, in other studies they do not correlate [22]. Hence, this study investigates reasons behind the mismatch in perceived and measured exposure whilst moving, explores how people experience air pollution and noise exposure and discusses if communication about exposure en route is needed. Specifically, if information about protective actions and healthier routes is increasing the recognition of own exposure and helpful for a pleasant commute. The aim of this study is to identify how to communicate route-based exposure to cyclists and pedestrians and discuss if exposure communication is worthwhile for supporting a healthy and pleasant commute in the city.

Many studies which provide air pollution or noise exposure feedback measured the pollution indoor using stationary measurement devices, only few measured and gave feedback on exposure outdoor and even less measured on-the-move and gave exposure feedback [23]. Specifically, studies measuring, reporting back and investigating perceived exposure of cyclists and pedestrians are rare (see literature review of [22, 23]). The few studies to date which provide cyclists or pedestrians with air pollution and related health information emphasize the potential of exposure communication for motivating users to take less polluted routes [24, 25]. However, recent literature reviews show both, successful outcomes (empowerment, protective practices, measurably lower pollution), but also identify resignation or helplessness as a result of exposure communication [23, 26]. For example, in some studies participants report sadness, fear or disappointment when receiving exposure feedback, in others interest and surprise [23]. Generally, it is not only about the form of the feedback, but about the pollution source and the feasibility to undertake protective practices [23]. Hence, it is important to research people’s needs and their coping ability when designing exposure communication and involve them in the development process. As shown by Riley et al. [26], few studies have included the public when developing exposure communication. Even less studies have involved cyclists and pedestrians. This study addresses this research gap by applying qualitative methods, specifically addressing commuters’ requirements, perceptions and practices. Focus groups were set up to understand if and how cyclists and pedestrians (also on their way to public transport) want to be informed about their exposure on commuting routes. Moreover, their exposure perceptions and protective practices were explored. Following a previous study in which participants engaged with air pollution and noise exposure on-the-move and simultaneously their exposure en route was measured [17], this study addresses three research questions:

Q1. In how far does a raised awareness regarding air pollution and noise exposure on commuting routes motivate people to protect themselves?

Q2. How can information on air pollution and noise pollution be designed to support healthy and pleasant mobility in urban areas?

Q3. Is information provision about exposure on daily (inevitable) routes a worthwhile strategy to support healthy and pleasant mobility?

In Sect. 2, the theoretical background is outlined. Sect. 3 presents the focus groups and the research design. In Sect. 4 the results are presented, specifically, participants’ motivation to protect themselves and their information preferences. This study specifically considers commuting routes, i.e., inevitable routes in the city for everyday purposes, and does not draw attention to leisure travel. In Sects. 5 and 6 the findings will be discussed and conclusions drawn.

## Theoretical background

Air pollution is a risk with a semantic pattern of perception, which it is hard to perceive: there is a complex cause-effect-relation and people need to consult information from third parties [27]. It is comparable to the risks perceived when smoking cigarettes: the invisibility of the threats and of long-term effects are similar to the invisible and long-term effects of air pollution [28]. Many studies on air pollution perception found a direct association between measurable air pollution and air pollution perception, only few studies did not find a correlation; however, from the studies which did not find a correlation, two referred to air pollution in or alongside traffic [22]. Hence, further investigating air pollution perception en route seems crucial. Studies on measurable noise pollution and perceived noise pollution found that high sound levels are not necessarily perceived as noise [19, 29, 30]. Generally, environmental health risks are more likely to be perceived by people if they are able to sense them [31]. The visual appearance of dust, the irritation to the eyes, nose or throat or the smell of exhaust fumes reinforce perception [31, 32].

### Protection Motivation Theory

Studies have shown that air and noise pollution are not always recognized as severe by people as they are measured [17, 19, 33], hence, people may not always undertake health protective practices regarding their exposure. Therefore, the question is how to inform about personal exposure and how to motivate people to develop coping strategies when moving around in the city. People do not always perceive air pollution or noise pollution as a threat, so it might be an issue of health care to motivate them to undertake protective practices. For researching how to motivate people to undertake healthier practices, the Protection Motivation Theory (PMT) [34] can be consulted. The PMT is applied for researching fear appeals and social cognitive variables which influence people’s intention to undertake protective actions [35]. It is a major theory in health behavior research and has also gained attention in environmental risk research [36, 37].

According to the PMT, a person’s motivation to protect oneself is influenced by two appraisal processes: the perceived threat appraisal and the coping appraisal [34, 35]. The threat appraisal considers how people estimate their vulnerability and how severe they evaluate the impact of stressors on their health [34, 38]. As for air pollution and noise exposure on-the-move it can be defined as:

- **Perceived vulnerability**

Perception of an individual towards her or his susceptibility to air and noise pollution, i.e., the perceived probability that air and noise pollution is harming while being on-the-move.

- **Perceived severity**

The perceived severity of air and noise pollution.

The coping appraisal refers to the degree to which a person believes his or her action can help to avoid the threat, involving self-efficacy and response efficacy [34, 38]. For air and noise pollution on-the-move it can be defined as:

- **Self-efficacy**

The belief that one is able to successfully enact the proposed avoidance strategies or protective actions regarding air pollution and noise pollution during commuting trips.

- **Response efficacy**

Expectancy that everyday exposure to air and noise pollution en route can be lowered through the recommended avoidance strategies or preventive actions.

The PMT was, for example, used to design information which was given to study participants to encourage healthy practices or used to create questionnaires on risk perception related to transport and Covid-19 [39]. The PMT is a valuable theory for predicting health-promoting practices [35] or designing interventions for healthy practices, such as mitigating personal exposure towards pollutants [23]. Specifically self-efficacy was found to strongly predict intentions to protect oneself from harm, even more than threat appraisal [35, 36]. The PMT is regarded as a valuable background for this study to understand which appraisals influence cyclists’ and pedestrians’ motivation to protect themselves from pollution en route. However, recent research has argued that the PMT is lacking consideration of social norms, which influence protection motivation as well [37]. For researching how to increase people’s threat appraisal and coping appraisal and linking it to environmental risk research, the concept for increasing environmental health literacy (EHL) is applied. Whereas PMT focuses more on the individual protection motivation [37], the EHL also addresses the collective dimensions of environmental health risks, drawing on civic life, environmental issues and wellbeing of others [40].

### Environmental health literacy

Becoming environmentally health literate can be regarded as “the fundamental capacity to understand and act upon the relationship between environmental exposure and health” (Stieb et al. [41], p. 2). Environmental exposure and hazards are often a community-wide problem which can hardly be tackled by the individual [37], thus, the concept of Environmental Health Literacy (EHL) is useful to address collective actions and civic engagement next to individual awareness [42, 43]. The concept of EHL can be used to structure information campaigns or exposure communication, for example, Johnston et al. [44] have applied the EHL for informing young people regarding their particulate matter (PM2.5) exposure, showing an increased EHL. According to Gray [42], EHL comprises three dimensions: (1) awareness and knowledge, (2) skills and self-efficacy and (3) community change. Finn and O’Fallon [43] further subdivide that into: recognition, understanding, application, analysis, evaluation and creation. In theory, the EHL enhances when a person progresses through each stage, gains knowledge and ultimately takes action [43]. The first stage encompasses the recognition and understanding that a pollutant is severe and impacts health. The EHL increases, resulting in an understanding of pollution and the capability to apply, analyse and evaluate data and ultimately, gaining skills to take action. With this increased self-efficacy people are then able to undertake health-protective actions and reduce exposure [42]. In the last step, people become capable of undertaking collective actions to reduce pollution, e.g., informing policy or becoming active in the community [42]. However, it should be noted that in order to move from one stage to the next, additional skills and knowledge are required. Also, certain competencies are needed for progressing in EHL, such as understanding feasible protective options, knowing strategies to take action or recognizing uncertainties [45]. When providing information that shall increase EHL, it should be considered that information on risks has to be beneficial for the person’s aims and be of interest for him/her, only then it stimulates an engagement with the information [27]. Moreover, practical or policy knowledge often needs to be incorporated to support the progressing from one stage to another, hence, integrating views from a multidisciplinary perspective is needed to create clear and actionable content [45]. Taking the concept of EHL for communicating air pollution and noise exposure in traffic could be a valuable approach for developing exposure communication strategies.

## Research design and methods

Qualitative research is scarce in the field of air pollution related health risk perception, yet, it offers great potential to gain in-depth understandings of air pollution perception and health patterns [46]. Hence, focus groups as a qualitative research method were chosen for this study. They are part of a broader research undertaken by Marquart et al. [17], comprising three phases. This article focuses on the third phase. The first two phases are individual studies but simultaneously served as preparations for the third phase, the focus groups. In the *first phase* “go- and ride-alongs” (qualitative interviews on-the-move) with cyclists and pedestrians in Berlin, Germany, were conducted and complemented by wearable sensors [17]. In this phase the participants were accompanied by an interviewer and asked about their perceived exposure and made aware of air pollution and noise pollution en route, applying a semi-structured interview guideline (see [18]). Meanwhile, particulate matter and noise levels were measured on-the-move using DylosLogger 1700 (particle number count) and a smartphone with external microphone, GPS and sensing application, based on Ueberham et al. [19] and described in [17, 18]. The particulate matter variations in numbers could be seen on the DylosLogger 1700 screen and could be discussed with the participants whilst on-the-move. The en route measurements were presented to the participants during the focus groups. In a *second phase* the same participants were asked to track their commuting routes individually on 3–5 days^1^ and complete a questionnaire about perceived exposure after each route taken using a tracking app (DLR MovingLab^2^). This aimed at increasing awareness regarding air and noise pollution on commuting routes and make the participants reflect on route choices. This served as a basis for the focus groups, which were done in a *third phase* and are subject of this article.

### Focus group approach

Focus groups are interactive discussions with a predetermined group of people who have shared experiences about a certain topic [47]. It is a valuable approach to allow the participants to create new solutions of a problem, discuss perceptions and opinions regarding a shared experience and create new ideas [47, 48]. The focus group approach is considered as valuable, because all participants shared experiences made during the go- and ride-alongs, the GPS tracking and their exposure en route was measured.

The recruitment took place via social media, newsletters, flyers, but also direct contact with offices in the city center and online neighborhood networks. This ensured that people with different life circumstances, educational background and commuting routes could be reached. As an incentive, participants were offered personal feedback on air and noise pollution. In total, 20 people participated in three focus groups, six till eight participants each. These 20 participants are out of the greater sample who had taken part in the first two phases. Two focus groups were held on-site, one online^3^. The focus group discussions took between 1 h 25 m and 1 h 41 m. An overview of the participants can be found in Appendix A.

### Procedure

The focus groups were held by a trained moderator using a semi-structured interview guideline, which comprised the following topics:

#### Shared experiences

Stimulating questions were asked underpinned by stimulus materials (pictures from the go- and ride-alongs, GPS-tracked routes of the individual GPS-tracking and a pollution perception ranking exercise). This should encourage an exchange and discussions about experiences and perceptions of air pollution and noise pollution (or other factors) en route. The questions specifically referred to the experiences made during to the go- and ride-alongs.

#### Feedback/Knowledge

Participants received feedback about their exposure during the go- and ride-alongs and also in the focus groups (brochure with measurement data), including measured exposure en route (text-based explanation of high exposure and low exposure situations, a map and graph with spatial variations) and information on feasible protective actions, text-based information about adverse health impacts and information channels such as WHO or Environmental Agencies (see Appendix B). Providing study participants with their monitored environmental exposure data enhances individual and community empowerment, can motivate to reduce or avoid exposure and improve environmental health literacy [49].

#### Information

Examples (pictures) of information sources, e.g. displays in the city or mobility apps, were presented, fueling a discussion about how air and noise pollution could be communicated. It was emphasized, that participants could creatively develop new and innovative ideas.

### Data analysis

All three focus groups were recorded and transcribed. For analysis a thematic coding was applied. This was done in an inductive-deductive approach to generate themes which are closely linked to literature and theory but still open to explore new and unforeseen topics [48, 50]. The program MAXQDA 2020 (version 20.4.1) for qualitative data analysis was used.

The analysis resulted in two themes developed deductively beforehand: (1) Protection Motivation and (2) information source and communication of risks. The categories of theme (1) were built deductively based on PMT, consisting of: (a) perceived vulnerability and (b) perceived severity of risks as well as statements regarding (c) self-efficacy and (d) response efficacy. Theme (2) followed deductively the concept of EHL, with the following categories: (a) information that support recognition and understanding, (b) information that support application, analysis and evaluation and (c) information that support creation and community change. During the focus groups another topic appeared, which was added later: (d) information denial. The categories were developed deductively based on the theory, the codes were then developed in a deductive-inductive approach out of the data (see Sect. 4). The transcripts were coded in two iterative rounds of coding.

## Results

The focus groups revealed that engaging with information about air and noise pollution en route as well as being made aware of one’s own exposure, as done during the go-/ride-alongs [16, 17], raised awareness. Participants’ perceived vulnerability and severity of risks and their motivation to protect themselves were enhanced. However, some factors lowered their perceived effectiveness of and ability to undertake protective actions: feeling powerless, the uncertainty and ubiquity of air and noise pollution and the importance of other factors for route choices (Sect. 4.1). The participants generated ideas for exposure communication, e.g., a healthy routing app, and demanded more community engagement. Some participants also denied receiving information on air and noise pollution, because they felt that the risk is too ubiquitous, indeterminate or it did not seem possible to protect themselves (Sect. 4.2).

### Risk perception and motivation to protect oneself

The engagement with data about personal exposure en route as well as the discussions about exposure during the go- and ride-alongs affected the perceived personal vulnerability and severity of air pollution and noise pollution. Also, a perceived low self-efficacy and a perceived low efficiency of protective actions were detected.

#### Perceived vulnerability and severity of risks

Participants are now more sensitive to air pollution and noise pollution from road or rail traffic. Engaging with their own personal exposure increased the perceived personal vulnerability on everyday routes. One participant thought the traffic situation or feeling unsafe were the source of her stress, whereas the interview made her realize that pollution smell and noise impact her wellbeing. The participants became confident in trusting own perceptions, if it was in line with the measurements. One participant started evaluating her exposure in other transport modes. Some participants described themselves as sensitive about pollution smells or noise and had felt vulnerable before, so the measurements underlined previous perceptions. However, being made aware of air pollution smells and sounds from traffic was not always reported as positive, some participants regretted they became alert:

*“One thing that has definitely increased is my awareness for air pollution. You have made me aware of the fact that you can smell it. And since then I smell it everywhere! And that annoys me, well, I don’t know if I’d rather not have known [laughs].”* (P16^4^)

*“My biggest eye-opener […] is that you have pushed me at some point: ‘how is it regarding the noise here?’ and only then I’m so much triggered to pay attention to the noise, which didn’t bother me at all before, and now it bothers me extremely. This is really negative. But the big worry is that there are more of these environmental influences […] saying, ‘oh yeah, probably something like that has been stressing me out all along, only I’ve never been able to say […] what it is.”* (P2).

Reporting back exposure can result in an increased risk awareness and increases perceived vulnerability, which may be a negative outcome from the participants view: Exposure feedback can draw attention to stressors that people were not previously aware of. This led to the fear that there are even more stressors of which one is unaware (as shown in the quote).

Participants reported that they learned that sometimes side roads with less air exchange had higher particulate matter levels than main roads with good air flow or how the number of particles varied depending on mode (car vs. bus), time (high pollution in evening hours) or distance to emitter. Altogether, participants reported increased knowledge. However, environments that were perceived to be healthy sometimes turned out to have higher pollution levels, resulting in anxiousness and uncertainties: for example, greenspaces or subways could have higher air pollution levels, even though these areas were perceived as rather healthy. This lowers the trust in own perceptions and pollution risks. Participants with children stated they fear the severity of air pollution when cycling or walking with their child and tried to increasing the distance to emitters at traffic lights or on the street.

However, sometimes participants did not feel at risk. Some said they were used to pollution in the city, did not smell air pollution, felt that cycling increased health anyway or did not feel health impacts:

*“I find it very difficult, for example, this question, to evaluate whether I felt a health burden. I can only say, no, I don’t have a shortness of breath. That would be a health burden for me. I can’t feel it. Of course, particulate matter and noise are a subliminal health burden, but at the moment I cannot define it like that.”* (P8).

*“I know about the particulate matter problems and so on, but, can I feel it? Can I measure it? Rather not. Big city.”* (P13).

For these participants air pollution and noise pollution seem to be a prevailing circumstance when living in a big city. The immediate effects of air pollution and noise pollution can hardly be felt. The knowledge that there is an underlying risk has increased, however, the immediate threat is not felt.

#### Self-efficacy and response efficacy

The participants also reported how they tried out and felt (not) able to protect themselves from air pollution and noise pollution. Table 1 summarizes their statements.

**Table 1:** Summary of the statements regarding coping appraisal. The particular topics as discussed by the participants during the focus groups and examples retrieved from the focus group discussion transcripts

Coping appraisal (deductively developed from PMT)	Topic discussed (inductively developed out of the data)	Description (examples)
Self-efficacy	Protective actions	Increased distance to emitter Cover nose/cover ear/hold breath
	Alternative routes	(Perceivably) less polluted routes are searched
	Alternative modes	Change towards less exposed modes (bicycle instead of subway)
	Emotion focused coping	Exposure is (mentally, in a psychological sense) suppressed to protect oneself
	Feeling powerless	Changing mobility practices is difficult Changing routes does not have desired effect (cf. response efficacy)
	Resignation / Prioritizing	Protective actions contradict with more important factors (e.g. safety, aesthetics, time, directness)
Response efficacy	Perceived health and wellbeing improved	Using headphones with calm music suppresses exposure Covering nose leads to a healthier feeling Changing mode is good for “body and soul”
	Refuse (and feeling that it is useless) to change route	Routes are already optimized Route changes are not possible (due to built environment) Changed routes have equally high exposure levels
	Importance of other factors	Changed route negatively impacts other factors (time, safety, aesthetics)
	Lack of political trustworthiness	Political actions are demanded to improve health/wellbeing en route, instead of individuals who have to find an efficient response to stressors

Some participants tried out new routes or changed their mode from train to bicycle. Others intended to wear a mask or pulled up a scarf, hold their breath, used headphones or covered ears. A common protective practice was to increase the distance to emitters, e.g., stopping in front of traffic lights or cars with visible or smelling exhaust fumes. Some protective practices made the participants feel that they had protected their health:

*“I’ve also pulled my scarf in front of my face [to lower air pollution inhalation], whether that actually helps at all? Hm… But I have the feeling that I can still breathe [in a highly polluted area].”* (P19).

On the contrary, participants often did not believe their route change improved health and wellbeing (response efficacy) or they felt unable to undertake protective practices (self-efficacy). Recurring topics were the feeling of powerlessness or resignation. This resulted partly from unexpectedly high exposure en route, the realization of its severity or the lack of understanding local pollution patterns. This led to a lack of confidence that individual actions can improve health, because pollution was perceived as ubiquitous. The resulting feeling of powerlessness was often associated with a lack of political trustworthiness:

*“But I do worry about what the results will be [the measurements]. Because everything changes so super slow or won’t change at all at the moment. […] Maybe I have to draw consequences at some point? No, I don’t want to move [walk/cycle] here anymore? Because I don’t want to stress my health for the next fifteen years?”* (P2).

*“So far, I haven’t really worried about it, on purpose. I decided for myself: I live in a big city, there is particulate matter pollution, it’s like that, I would be happy if it were lower, but I accept that as a marginal condition.”* (P13).

The lack of confidence in planning and policy led to resignation and made people consider to move away from the city to stay healthy. Generally, suppressing exposure was a common action to deal with pollution risks on inevitable routes. Some reported resignation, since there was no other option than the polluted route:

*“I actually looked for alternatives a long time ago and then I gave up at some point, because there were none. No better alternatives. There are alternatives, but only worse ones. Well, what I do is already what I can do and that worries me, of course. Of course, I don’t want to get sick just because the politicians haven’t yet realized [that the pollution is too high]. I think that’s really bad…”* (P1).

*“I checked whether I can cycle here [points at a parallel street of the main road on a map], that is parallel to the main road, so to speak. […] but these are all closed neighborhoods, which means I ultimately end up back [on the main road] and somehow it doesn’t make sense. So, I thought at this point: rather a short, intense exposure than a longer medium one.”* (P6).

These quotes show that receiving information about high exposure may lead to a feeling of injustice, worries, helplessness, ignorance or acceptance, but not necessarily to an increase in self-efficacy. Some participants complained about lack of alternatives: the infrastructure often guides them along main roads with motorized traffic and perceivably less polluted residential areas were often unsuited to cycle. Moreover, routes were often optimized considering personal preferences, whereas air pollution and noise pollution were not (yet) priorities in route choices. Other factors were more decisive: time/directness, relaxing and not concentrating, quietness, aesthetic, livability or shops, excitement or safety. As a result, some participants did not want and did not feel able to change routes. Generally, most of the participants demanded a built environment which offers routes that are at the same time low polluted, pleasant and safe. One participant summarized these worries:

*“But, the problem is, if we have no choice but to take this route, then the information is of no use to me. So, you have to get to the root [of the problem].”* (P4).

Hence, if a person is not able to change towards a healthy route, information provision is not considered as valuable.

### Information needs

**Fig. 1:**
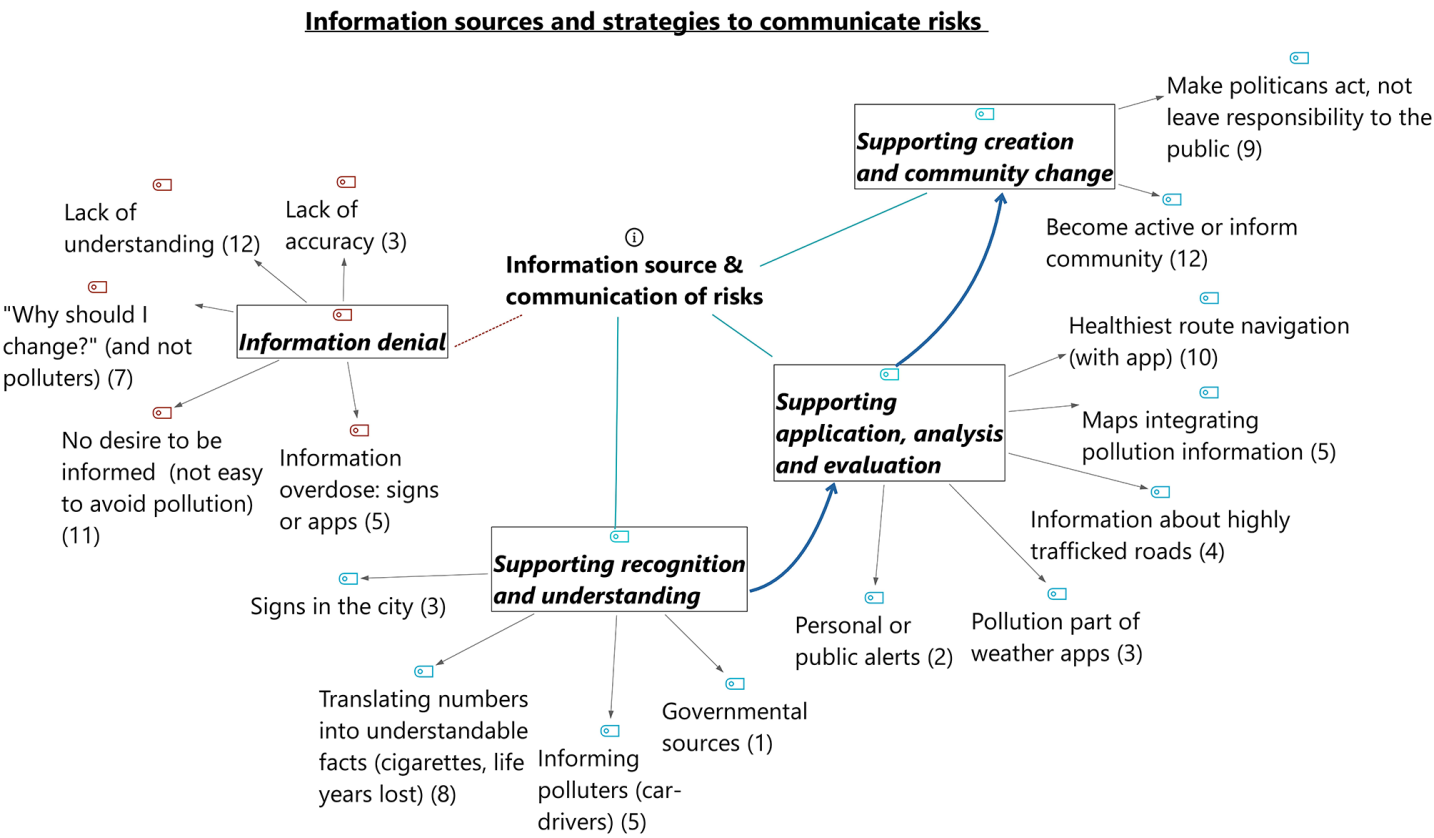
Overview of the codes retrieved during the thematic coding. The main codes are based on the main steps of increasing Environmental Health Literacy (blue), which built on one another. It starts with recognition and understanding, then leads to application, analysis and evaluation and ultimately results in creation and community change. The retrieved codes were allocated to the suitable EHL stage. Statements regarding information denial were coded separately (red).

The participants discussed how they want to be informed. Their statements addressed all stages of EHL, but they sometimes also refused to receive information at all. Figure 1 provides an overview.

#### Information supporting recognition and understanding

Information supporting recognition and understanding aims at informing people, who have not encountered with the topic yet. Participants discussed solutions such as displays in the city showing daily pollution, which was part of the focus groups’ stimulus material. This would also inform polluters. Yet, it is hard to make sense of the numbers of city-wide displays. Measured exposure should be translated into something easy to understand, e.g., life years lost, comparing it to cigarettes smoked or pictures of health impacts:

*“[…] So that you would know, hey, how many cigarettes would I smoke on my way there […] If I knew, I would smoke one versus three cigarettes on my way to work, I might decide to take another route with only one cigarette.”* (P18).

Risks could also be translated into health burdens, e.g. headaches, problems with breathing/lungs or high stress levels associated with air and noise pollution exposure. This would specifically address the threat appraisal (see PMT). As discussed, however, the coping appraisals might be more influential [35, 36].

#### Information supporting application, analysis and evaluation

Healthiest route navigation was discussed the most for supporting individual mobility. Many participants desired an app that integrates current pollution levels, but also suggests alternative routes with less pollution. The “healthiest route” as an option next to, e.g., “fastest”, “least cobble stones” or “most beautiful” was recommended. Participants who know the city well preferred maps with real-time air pollution and noise pollution to choose their own routes. However, as pointed out by some participants, the healthiest routing would demand for small-scale real-time pollution data. A suggestion to address this issue was an app with information on real-time information about the traffic volume on a street, which is associated with noise and air pollution.

#### Information supporting creation and community change

In the course of the focus group discussions the participants started questioning why they only talk about passively receiving information, instead of actively changing the situation. Taking part in the study and becoming aware of air and noise pollution resulted in a strong sense of activism and public engagement. The participants suggested a range of activities for the community: informing others or the public, integrating the topic in schools or adult education, supporting local initiatives, or even suing the state for not preventing health impacts. Participants also became multiplicators:

*“I definitely walked through the neighborhood much more consciously and also talked with many people about particulate matter pollution. And talked with my friend about why it’s so dirty on our first floor, because maybe it’s also dusty due to the street in front of the door and so on. So, there’s a lot of things that have continued [after the interview].”* (P19).

Next to public engagement, the participants demanded political actions. Some called for more political actions to create healthy routes, instead of leaving the responsibility to search healthy routes to the cyclists and pedestrians. They concluded that they did not only want to be informed where pollution is, but get to the root of the problem and influence policy and planning.

#### Information denial

During the focus groups the participants wondered why they, as the most environmentally friendly road users, should change their routes to protect themselves from pollution caused by motorized traffic (“*why should I change?*” (P10)). Less polluted routes were often perceived longer or less pleasant. Some of the participants reported anger and resignation that they have to be responsible for travelling in a healthier way by individually changing their route. Some participants feared that information provision, e.g., smartphones, might shift the focus away from the actual problem:

*“That is interesting. That makes me think… That I automatically remain in the position of the weaker road user and say: Gosh! An app like that is great! But actually,[…] we have forgotten the main topic! Namely, the elephant in the room [idiom]. We must first take away space from the cars, […] [space], which is already allocated [to the cars]. You have to give more space to cyclists, cyclists and pedestrians.”* (P9).

This resulted in information denial and refusal of receiving information. Many participants concluded they did not necessarily want to be informed. Moreover, receiving information about pollution on inevitable routes could cause stress and hence, cause health problems and lower wellbeing:

*“Actually, I don’t want to be informed about it at all, because in the end, it makes me sick. If I don’t know, I have fewer problems with it.”* (P15).

*“But [I do not want information] all the time [from] displays or so, because then, I think, I will always get in a worse mood and could also… […] then it would totally piss me off and that doesn’t do me any good, if I am upset about it all the time.”* (P20).

Some participants reported they feel already overloaded by information, e.g., from apps or signs. Receiving additional pollution information on-the-move could increase stress, especially if the information appears when a person already rides through highly exposed areas and cannot avoid it (“*that would stress me out rather than do me any good”* (P17)). .

Finally, protecting against long-term effects of air pollution or noise pollution can contradict with the short-term desire of a comfortable, short and pleasant route. Information which is contradictory to one’s own feelings and belief may lead to inaction. One participant summarized her confusion:

*“If I perceive something as more pleasant, should I rather take this route or should I, based on the findings that we have, say: well, then I take rather the route which does not feel so good, but is actually quieter and therefore may not be a threat to my health in the long-run? Well, are there any suggestions, how we should behave? Should I rather choose according to my own perception or should I actually act according to the data?”* (P20).

These contradictions will be discussed in the following.

## Discussion

The aim of this study was to identify how to inform cyclists and pedestrians about their noise and air pollution exposure on daily commuting routes in the city. First, communicating personal exposure to air pollution and noise pollution can empower individuals to undertake protective practices and leads to community engagement, but at the same time can also result in resignation and the feeling of powerlessness (Q1). Second, the discussion will highlight opportunities to inform about air pollution and noise pollution to support healthy and pleasant mobility (Q2), discussing that also enjoyable and pleasant route suggestions could be integrated. Finally, the question if exposure information is worthwhile to support healthy and pleasant mobility will be discussed (Q3), elaborating on participants’ call for political actions and their engagement in the community.

### Empowerment vs. resignation

Addressing research question Q1, receiving information on exposure increased knowledge and raised awareness: Pollution smells or traffic sounds were now perceived en route. Health impacts (e.g. headache, stress) were put in relation to the stressors. Yet, increased awareness also has its downsides.

In line with other studies, perceptions did not always match with measurements [17, 19, 29, 51] and participants partly appreciated the knowledge gain and felt confident to trust their perceptions. Reporting-back exposure measurements was regarded as positive and people are eager to receive “their” results [49, 52]; it usually increases EHL and has the potential to tackle urban air pollution [26, 42]. As for noise, research reporting back noise pollution data to the public is rather scarce [23]. People are often not aware of traffic noise and lack actions to avoid it [53, 54]. This is similar to the participants in this study. However, if people get more involved in noise pollution monitoring, they show an increasing knowledge of noise pollution in the city and a higher awareness of pleasant and unpleasant soundscapes [55]. They may be able to evaluate and integrate quieter route sections into their daily commute. Hence, participatory noise pollution monitoring has great potential to empower people and raise awareness [56, 57]. Knowing about noisy and quiet areas, such as green spaces, can decrease the perceived adverse noise pollution effects [58]. Some of the participants have already undertaken protective practices to lower noise pollution by using headphones to listen to music or podcasts (see Sect. 4.1.2), however, this protective practice may also affect perception of sounds (e.g. cars) which act as an attentional trigger and are decisive for safe cycling or walking [59]. Some cyclists compensate that by e.g. looking around more often, using only one earbud or turning down the music [59], however, safety issues need to be considered for this protective practice. Summarizing, information on personal exposure can empower laypersons to take action and raise awareness.

Conversely, the increased awareness of a risk, which had been suppressed before, was sometimes regarded as negative. After becoming aware of smelling polluted air or hearing traffic sounds some participants felt unable to ignore it anymore. Annoyance or stress increased. Some participants wished they would never have known (Sect. 4.1). Some air pollution studies link air pollution perceptions with (self-reported) health symptoms [22]. Perceiving odor of air pollution can lead to annoyance and trigger actual health symptoms, specifically if the odor is perceived as unpleasant [60, 61]. This is similar to noise: if a person perceives noise and feels distressed, the noise causes psychological stress, whereas the measured noise pollution itself may not significantly influence psychological stress [62]. Exposure information about stressors, which had been successfully suppressed before, can backfire and result in negative feelings, resignation and psychological stress. Nevertheless, the measurable exposure has an assessable health impact in the long term [1, 2, 7], even if it is not perceived in situ. Exposure information has therefore to be designed with caution, limiting its potential to result in psychological stress. This can be done by including information about feasible and existing protective measures.

### Barriers to take action: uncertainty and ubiquity

Having feasible options to protect oneself is important. Exposure information is of no need if there are social or environmental barriers which prevent behavioral adaptations [26]. For example, built environmental factors can hinder people to take routes away from pollution or it can be the case that other factors are more decisive for route choices (e.g. safety, aesthetics, distance, time) (Sect. 4.1.2).

A lack of understanding can also lower self-efficacy. Similar to Noel et al. [46], the link between air pollution and one’s health is often shaped by uncertainty. Participants want pollution numbers to be translated into something relatable, e.g. life-years lost or cigarettes smoked (Sect. 4.2.1). Ambient air pollution is complex and impacted by a variety of factors (e.g. wind), hence, it does not always match with people’s expectations (e.g. green spaces have high pollution (“green-is-clean assumption”) [63]). Presumably healthy routes are not necessarily pollution-free. This may result in doubts that healthy alternatives exist at all [64]. Relatable information is even more difficult for noise pollution on-the-move. High dB(A) levels do not necessarily represent sounds perceived as noise: the situational context, the activity performed and the transport mode impact if a sound is perceived as noise [18, 30, 62]. The reported discrepancies of measured and perceived noise on-the-move prove evidence [18, 19]. Providing real-time dB(A) measurements may not be a useful indicator, since it may not reflect actual noise annoyance. This raises the question how to lower exposure to air and noise pollution, especially if its spatial patterns are complex and the situational context decisive.

Firstly, it is recommended to prioritize planning of less polluted routes and consider other factors (aesthetics, safety, distance) when planning for cyclists and pedestrians. Only then healthy routes are an option, and information is useful. Secondly, information on air pollution and noise pollution should be relatable and address uncertainty and ubiquity, e.g., by improving real-time data with a city-wide monitoring network. Lastly, it may not be worthwhile to provide people with exposure information at this stage if they cannot change their routes. This will be elaborated in the following.

### Informing about air pollution, noise pollution and pleasant routes

Exposure information should have a positive framing, trigger people emotionally, provide relevant information, communicate the co-benefits of behaviors and support undertaking the action [26]. Technical information has to be enriched with emotional triggers to be effective [45]. Studies reporting back exposure which led to protective practices exist: they included storytelling-approaches or workshops with the community next to measurement data [44, 65]. This empowered and created a feeling of ownership over the measurement campaign [44, 66]. This is similar to this study’s findings: participants felt empowered by being involved in the research process.

Several possibilities to inform commuters were identified. Information should be more than just numbers [67]. Pollution measurements could be translated into illustrations or integrated in a healthy routing app. As shown by Marquart and Schuppan [68], a healthy routing app could draw on the PMT and integrate other health-related factors, among air pollution and noise pollution. As health considerations are an important motivation for people to walk or cycle [69], a healthy routing app might be specifically interesting for cyclists and pedestrians. However, as this study has shown, a healthy routing app alone may not be worthwhile, because other decisive factors influence a pleasant commute.

As shown in this and previous research [17], sensory awareness (seeing and smelling greenery or water, aesthetics, interesting sights) and social cues can even balance negatively perceived pollution. Experiencing positive emotions and wellbeing can results in an increased physical health in general [70, 71]. Consequently, a healthy route planning app could include pleasant trip characteristics alongside pollution data. It could include social cues, such as bars and cafés, urban gardening and allotments, playgrounds or pedestrian areas, greenery and water or interesting ‘highlights’ (e.g. landmarks) along the route (see Marquart et al. [17]), next to pollution. These could be combined with factors such as directness or safety, shown to be decisive for cycling as well [69, 72]. Moreover, cyclists and pedestrians know best which factors increase their personal wellbeing along routes. With community mapping people could collectively collect knowledge, emotions and experiences of their routes and share them with peers [73]. Adding this to route planning apps could provide collective knowledge on pleasant or (hidden) less polluted routes. Exchanging subjective mobility experiences via smartphone apps can enhance the own trip experience [74]. There is no need to solely rely on general route planning apps, but include and share collective information on pleasant, less polluted routes with peers by including a community mapping approach in mobility apps.

### Political actions and community engagement

Concerning Q3, the information denial stated by the participants shows that exposure information on daily (inevitable) routes may (at the moment) not be worthwhile. Some participants request that health gets a higher priority in planning decisions. This is also reflected by Ramirez et al. [67], who argues that air pollution communication concentrates too much on individual risk-fighting behavior and does not address the “structural factors” creating pollution. The increased knowledge of air pollution and noise pollution inspired some participants to raise the awareness about these stressors in their community. This is in line with other report-back studies (e.g. Tomsho et al. [75]). Facing negative environmental conditions is a motivator for environmental protest [76]. Raising awareness for air pollution and noise exposure can result in community engagement and activism [76]. Mobility apps, as the one proposed here, could integrate community mapping and communicate perceived air pollution or noise pollution to decision-makers, hence, be a valuable cornerstone for urban planning [52, 73].

### Limitations

First, it should be noted that the sample may consist of an interested and rather concerned group in terms of environmental concern, who may – at least partly – be educated regarding pollution problems. As shown in previous research [77], people who feel annoyed by air pollution are usually those who are worried about it. Hence, they may participate more likely in air or noise pollution research. This rather “special interest groups”, who can be dominant in participatory processes, may lead the discussions and put their interests in the focus [78, 79]. Hence, less dominant participants or other vulnerable groups, e.g., people with diseases (e.g. asthma) or children, may not participate in an extensive participatory research as this one. Future research could specifically take vulnerable populations into account [75], particularly concerning exposure in traffic, but also focus on having a diverse educated group of people when researching EHL and exposure communication. Second, taking the EHL as a framework for exposure communication can be fruitful, yet, the knowledge, skills and competencies of the targeted group need to be considered when designing exposure information. Not all people start from the same stage. Considering pre-existing knowledge, skills and competencies of the targeted group is crucial. Third, the focus groups do not give conclusions in statistical terms. The qualitative approach aims at exploring the research subject under investigation in depths and how it is constituted, rather than investigating its statistical characteristics [80]. Qualitative approaches can help to better understand environmental risk perception [37, 46] and give deep insights into how people perceive air or noise pollution, how they protect themselves and are helping to identify communication needs and planning requirements.

## Conclusion

This article contributes to people-centered exposure communication research by exploring how cyclists and pedestrians want to be informed about a less polluted and pleasant commute. First, it was shown that providing exposure information should be centered around commuters’ needs, their coping abilities and knowledge in order to avoid information denial. Addressing theories like Protection Motivation Theory (PMT) or concepts like Environmental Health Literacy (EHL) when designing information is recommended. Second, the ubiquity and uncertainty of urban air pollution and noise pollution together with the inevitability of daily commute can result in a feeling of helplessness and resignation, raising the question in how far exposure communication is worthwhile for commuting trips. Integrating air and noise pollution information in a mobility app enriched with other pleasant route aspects (“pleasant routing app”) and participatory approaches is promising. Future research could develop and test an app like this, also applying quantitative methods such as surveys or GPS tracking. Ultimately, policy and planning should not leave the responsibility to the exposed road users in finding healthy routes, rather should environmentally-friendly mode users be protected against health-impacting air pollution and noise pollution by implementing planning measures. The perceived exposure, social cues and sensory awareness (greenery and water, aesthetics and interesting urban form) should receive attention in informing and planning for cyclists and pedestrians [17]. An app, which integrates pollution, pleasant route environments and addresses participatory approaches could support healthy, pleasant and pollution-free mobility in urban areas.

## Data Availability

The data of the study will be made available upon reasonable request.

## Appendix

**Appendix A: Overview of the participants**

**Table:**

Participant No.	Age	Female / male	Modes of transport available	Commuting mode	Employment status
Focus group 1					
P1	55	f	Bicycle, car-sharing, public transport ticket	Bicycle	Full-time
P2	30	m	Car, bicycle	Bicycle	Part-time (flexible)
P3	n/a	m	Bicycle, public trasnport ticket	Bicycle	Paternity leave
P4	59	f	Bicycle, scooter sharing, public transport ticket	Walking + public transport	Self-employed
P5	25	m	Bicycle, public transport ticket	Bicycle, walking + public transport	Student
P6	24	m	Bicycle, public transport ticket	Bicycle, walking + public transport	Part-time (flexible), student
P7	42	f	Bicycle, car-sharing	Bicycle	Full-time
Focus group 2					
P8	51	f	Car, bicycle	Bicycle	Part-time
P9	58	m	Bicycle, car-sharing, e-scooter-sharing, public transport ticket	Bicycle + public transport	Full-time
P10	50	m	Car, bicycle, public transport ticket	Bicycle, walking + public transport	Full-time
P11	51	f	Car, bicycle, car-sharing, bicycle-sharing	Bicycle	Full-time
P12	37	f	Bicycle	Bicycle	Part-time (flexible)
P13	45	m	Bicycle, car-sharing, bike-sharing	Bicycle	Full-time
Focus group 3					
P14	28	f	Bicycle	Bicycle	Student
P15	61	m	Car, bicycle, motorbike	Bicycle	Full-time
P16	29	m	Bicycle, car-sharing, scooter-sharing, public transport ticket	Bicycle	Part-time (flexible), student
P17	31	m	Bicycle, car-sharing, bicycle-sharing	Bicycle	Part-time (flexible)
P18	31	f	Bicycle, car-sharing, public transport ticket	Bicycle	Full-time
P19	30	f	Bicycle, car-sharing, bicycle-sharing, public transport ticket	Bicycle, walking + public transport	Part-time (non-flexible), student
P20	33	f	Bicycle, car-sharing, bike-sharing, scooter-sharing	Bicycle	Part-time (flexible), self-employed

**Appendix B: Example of two pages from the reporting-back brochure used as stimulus material during the focus groups: information on health impacts of exposure and potential protective practices (left) and an example of reported back noise measurements (right; translated from German to English for this article; the measurement data is made blurry for privacy reasons)**.

## Footnotes

1 Due to the outbreak of the Corona pandemic in early 2020, two groups did not commute daily to work anymore and hence, did not track their routes on five days.

2 www.movinglab.dlr.de.

3 Due to the Corona pandemic in late 2020, the third focus group was held online in November 2020 and the second focus group was in a hybrid format with two participants participating online and the others on-site (September 2020). The first focus group took place before the Corona pandemic in December 2019.

4 P# refers to „Participant ‘number’“ (see Appendix). Note that all interview statements in the paper are translated by the author from German to English.
